# How has implementation been incorporated in health technology assessments in the United Kingdom? A systematic rapid review

**DOI:** 10.1186/s12961-021-00766-2

**Published:** 2021-08-18

**Authors:** Robert Heggie, Kathleen Boyd, Olivia Wu

**Affiliations:** Health Economics and Health Technology Assessment (HEHTA), Institute of Health and Wellbeing, University of Glasgow, 1 Lilybank Gardens, Glasgow, G12 8RZ UK

**Keywords:** Implementation, Economic evaluation, Mixed methods, Health technology assessment

## Abstract

**Objectives:**

Health interventions in a clinical setting may be complex. This is particularly true of clinical interventions which require systems reorganization or behavioural change, and/or when implementation involves additional challenges not captured within a clinical trial setting. Medical Research Council guidance on complex interventions highlights the need to consider economic evaluation alongside implementation. However, the extent to which this guidance has been adhered to, and how, is unclear. The failure to incorporate implementation within the evaluation of an intervention may hinder the translation of research findings into routine practice. This will have consequences for patient care. This study examined the methods used to address implementation within health research conducted through funding from the National Institute for Health Research (NIHR) Health Technology Assessment (HTA) programme.

**Methods:**

We conducted a rapid review using a systematic approach. We included all NIHR HTA monographs which contained the word “implementation” within the title or abstract published between 2014 and 2020. We assessed the studies according to existing recommendations for specifying and reporting implementation approaches in research. Additional themes which were not included in the recommendation, but were of particular relevance to our research question, were also identified and summarized in a narrative synthesis.

**Results:**

The extent to which implementation was formally incorporated, and defined, varied among studies. Methods for examining implementation ranged from single stakeholder engagement events to the more comprehensive process evaluation. There was no obvious pattern as to whether approaches to implementation had evolved over recent years. Approximately 50% (22/42) of studies included an economic evaluation. Of these, two studies included the use of qualitative data obtained within the study to quantitatively inform aspects relating to implementation and economic evaluation in their study.

**Discussion:**

A variety of approaches were identified for incorporating implementation within an HTA. However, they did not go far enough in terms of incorporating implementation into the actual design and evaluation. To ensure the implementation of clinically effective and cost-effective interventions, we propose that further guidance on how to incorporate implementation within complex interventions is required. Incorporating implementation into economic evaluation provides a step in this direction.

## Contribution to the literature

- Current guidance on developing and evaluating complex interventions recommends that implementation should be considered as part of a cyclical process—development, feasibility/piloting, evaluation, and implementation.
- There are no formal guidelines or frameworks for how implementation can be incorporated within a holistic evaluation of a health technology.
- Our review sought to identify if, and how, implementation has been taken into account in NIHR HTA research over the last 6 years.
- Our review found that, although informal and inconsistent, methods are available to address implementation. Economic evaluation provides a set of tools which can aid implementation. However, further research and formal guidance are required to ensure the translation of research findings into clinical practice.

## Background

Clinical research findings are often challenging to implement into routine clinical practice**.** This is particularly true of complex interventions which require significant system reorganization, behavioural change, or when implementation involves additional challenges which are not captured within a clinical trial setting. To ensure potentially beneficial research findings are effectively translated into routine clinical practice, one needs to consider implementation.

There are many reasons why a potentially promising health technology observed in a clinical trial setting may not translate into an improvement in patient outcomes in a routine clinical setting [1]. Among these is the consideration of the barriers presented by costs and consequences not observed in a trial setting. The underuse of potentially beneficial health interventions has consequences in terms of potential patient benefit forgone [2].

Given that limited resources are available to generate patient health outcomes in a publicly funded healthcare system, it is necessary to consider both the clinical effectiveness and cost-effectiveness of health technologies. This includes the choice of how, and indeed whether, to implement a health technology [3, 4]. Economic evaluation provides a tool by which researchers can determine not only whether or not a health technology should be implemented and the extent of implementation required, but also the conditions under which a technology would be expected to be cost-effective. In the realm of complex interventions, it may also be necessary to consider the cost-effectiveness of systems-level changes in healthcare provision and the cost-effectiveness of a single technology given alternative configurations of the healthcare system or clinical pathway.

Economic evaluation plays an increasingly crucial role in the evaluation of health technologies. However, despite this, economic evaluation rarely considers explicitly the challenge of implementation. In recent years, some methodological tools have been developed which seek to bridge the gap between economic evaluation and implementation science [5–8]*.* Economic evaluation can potentially aid implementation in two ways. It can either be used to compare alternative implementation strategies—i.e. by considering the costs and consequences of implementation strategy X, compared with Y [5]. Alternatively, implementation challenges can be incorporated within the economic evaluation of a technology—i.e. by adopting a mixed-methods approach to economic evaluation [6–8].

Although typically the reserve of population health studies, complex interventions are increasingly relevant to interventions in a clinical setting. The line which distinguishes a “simple” from a “complex” intervention is blurred. Indeed, some argue that the distinction relates to the choice of research question, rather than the intervention itself [9, 10]. From the perspective of a health technology assessment (HTA) body, whose remit is to consider the clinical effectiveness and cost-effectiveness of a health intervention, alongside equity and other social concerns, it could be argued that all interventions should be evaluated as complex interventions.

The importance of implementation is recognized in current Medical Research Council (MRC) guidance which highlights four phases for the assessment of complex interventions in a “cyclical sequence”: development, feasibility/piloting, evaluation, and implementation [11]. Furthermore, as part of the implementation element of a complex intervention, the MRC guidance highlights dissemination, surveillance and monitoring, and long-term follow-up as the key issues to consider—all following the evaluation process. There is no discussion of how implementation can be used to inform the evaluation process. The MRC guideline update is currently underway and will address additional elements including early economic evaluation alongside the consideration of implementation [12].

The National Institute for Health Research (NIHR) is the largest funder of health-related research in the United Kingdom. The need to undertake an economic evaluation of a health technology is a core component of the NIHR Health Technology Assessment (HTA) programme. Therefore, this rapid review sought to examine how implementation has been incorporated into NIHR HTA research over the past 6 years.

## Methods

We conducted a rapid review, using a systematic approach [13], to examine how implementation has being taken into account within NIHR HTA research. We applied the Proctor et al. (2012) checklist to identify how issues relating to implementation had been included within each study [14, 15]. In addition, we identified additional themes that are relevant but not captured within the Proctor et al. (2012) checklist. A narrative synthesis was undertaken using these key themes to evaluate and discuss the identified studies.

### Criteria for inclusion of studies

We included NIHR HTA monographs published over the period September 2014—September 2020. All monographs which contained the word “implementation” within the title or abstract were included for review. Details of the search terms are given in Table 1*.* All monographs obtained from the search were included in the review. No exclusions were made based on participants, interventions, comparisons, outcomes, or study design. As the purpose of this review was to evaluate how implementation has been incorporated into all studies identified in the review, no quality assessment of the identified studies was required.

**Table 1:** Search terms used in literature review

#	Search terms	Results
1	health technology assessment winchester england.jn	
2	implementation.mp. [mp = title, abstract, original title, name of substance word, subject heading word, floating sub-heading word, keyword heading word, organism supplementary concept word, protocol supplementary concept word, rare disease supplementary concept word, unique identifier, synonyms]	171,395
3	limit 2 to (abstracts and yr = "2014 -Current")	65,022
4	1 and 3	42

### Database searched

We searched the NIHR HTA database via Medline.

### Data extraction

All monographs were retrieved from Medline and exported to Endnote X7.0.2. They were initially reviewed, and data were extracted by one researcher. For the purpose of validation, a random sample of 10% of the monographs were subsequently reviewed independently by two additional researchers.

### Data synthesis and presentation

All monographs were reviewed and assessed according to the Procter et al. (2012) checklist. This checklist was designed to provide guidance for researchers planning an implementation study. It contains a list of criteria which the authors recommend should be addressed within a study which aims to evaluate implementation. It is based on a review of successful implementation study research grants and the broader literature on implementation studies. Other checklists have been used when assessing the quality of studies used to inform implementation [16, 17]. However, the focus of these checklists was on the quality of survey methods used to inform implementation, rather than a focus on how implementation had been incorporated into the study. To date, we are not aware of any commonly accepted tool for incorporating implementation into the development and evaluation of a study. For this reason, we believed the Proctor et al. (2012) checklist served as a suitable tool for assessing the extent to which implementation issues have been incorporated within the studies included in our review. The key components relating to implementation that we used to critique the studies in our review, based on the Proctor et al. (2012) checklist, are identified in Table 2.

**Table 2:** Proctor et al. (2012) checklist

	1. Care or quality gap	2. Evidence-based practice	3.a Theoretical justification	4. Stakeholder engagement	5. Setting	6. Implementation strategy	7. Team expertise	8. Study design	9. Measurement	10. Policy/funding environment
Campbell et al. (2014)	✓	✓	✓	×	×	×	×	✓	×	×
Hood et al. (2014)	✓	✓	✓	✓	×	×	✓	✓	×	×
Livingston et al. (2014)	✓	✓	✓	✓	×	×	✓	✓	×	✓
Bonell et al. (2015)	✓	✓	✓	✓	×	✓	✓	✓	✓	✓
Freeman et al. (2015)	×	✓	✓	×	×	×	×	✓	×	×
Guthrie et al. (2015)	×	✓	✓	✓	×	×	×	×	×	×
Michie et al. (2015)	✓	✓	✓	✓	×	×	✓	✓	×	×
Richardson et al. (2015)	✓	✓	✓	×	×	×	✓	✓	×	✓
Bailey et al. (2016)	×	✓	✓	✓	✓	×	✓	✓	×	✓
Field et al. (2016)	×	✓	✓	✓	×	×	✓	✓	×	✓
Fortnum et al. (2016)	✓	✓	✓	✓	×	×	✓	✓	×	×
Freeman et al. (2016)	✓	✓	✓	×	×	×	×	✓	×	×
Jackson et al. (2016)	×	✓	✓	✓	✓	×	✓	✓	×	✓
Parry et al. (2016)	×	✓	✓	✓	×	×	✓	✓	×	×
Paton et al. (2016)	×	✓	✓	×	×	×	✓	✓	×	✓
Tufail et al. (2016)	✓	✓	✓	×	×	×	✓	✓	×	×
Whitaker et al. (2016)	✓	✓	✓	✓	×	×	✓	✓	×	✓
Birrell et al. (2017)	×	✓	✓	×	×	×	×	×	×	×
Flowers et al. (2017)	×	✓	✓	×	✓	×	✓	✓	×	✓
Melendez-Torres (2017)	✓	✓	✓	×	×	×	×	×	×	×
Snooks et al. (2017)	×	✓	✓	✓	×	×	✓	✓	×	✓
Soomro et al. (2017)	✓	✓	✓	✓	✓	×	×	✓	×	×
Thomas et al. (2017)	✓	✓	✓	✓	×	×	✓	✓	×	×
Watson et al. (2017)	×	✓	✓	✓	×	×	✓	✓	×	×
Waugh et al. (2017)	✓	✓	✓	×	×	×	×	✓	×	×
Welton et al. (2017)	×	✓	✓	×	×	×	×	✓	×	×
Williams et al. (2017)	✓	✓	✓	✓	×	×	✓	✓	×	×
Avenell et al. (2018)	✓	✓	✓	×	✓	×	×	✓	×	×
House et al. (2018)	×	✓	✓	✓	×	×	✓	✓	×	×
McClurg et al. (2018)	✓	✓	✓	✓	×	×	✓	✓	×	×
Paleri et al. (2018)	✓	✓	✓	✓	×	×	✓	✓	×	×
Peron et al. (2018)	×	✓	✓	✓	✓	✓	✓	✓	✓	×
Richards et al. (2018)	×	✓	✓	✓	×	✓	✓	✓	✓	×
Saramago et al. (2018)	×	✓	✓	×	×	✓	✓	✓	✓	×
Seguin et al. (2018)	×	✓	✓	✓	✓	×	✓	✓	×	✓
Allan et al. (2019)	✓	✓	✓	✓	×	×	✓	✓	×	×
Griffin et al. (2019)	×	✓	✓	✓	✓	×	✓	✓	×	×
James-Roberts et al. (2019)	×	✓	✓	✓	×	×	×	✓	×	×
Madan et al. (2019)	✓	✓	✓	✓	×	×	✓	✓	×	✓
Simmonds et al. (2019)	✓	✓	✓	✓	✓	✓	✓	✓	✓	✓
Francis et al. (2020)	×	✓	✓	✓	×	×	✓	✓	×	×
Surr et al. (2020)	✓	✓	✓	✓	✓	✓	✓	✓	✓	✓

Due to the limitations associated with the use of the Proctor et al. (2016) checklist for the purpose of this study, a narrative synthesis was used to identified additional themes relevant to the issue of implementation, but not captured within the Proctor checklist [18, 19]. We grouped “themes” not captured within Proctor. These themes were identified by the three study authors as themes which can aid the incorporation of implementation within economic evaluation. We identified and presented these themes alongside each study in matrix form in Table 3. As there was no “standardized metric” among studies, meta-analysis of results was not appropriate. We evaluated how the inclusion or exclusion of these additional themes served to hinder or facilitate the incorporation of implementation within the studies. We discussed heterogeneity of our results in terms of the consistency of approach and any pattern of change over time. Limitations to our review, such as databases searched and themes identified, are discussed in the Discussion section.

**Table 3:** Additional themes not captured in the Proctor et al. (2012) checklist

	Intervention	Study type	Process evaluation	Barriers and facilitators	Quantitative evaluation of implementation	Included economic evaluation	Clear recommendations for implementation	Implementation as future work
Campbell et al. (2014) (28)	Cardiac MRI in ischaemic cardiomyopathy	Review and model	×	✓	×	✓	×	✓
Hood et al. (2014) (29)	Probiotics for antibiotic-associated diarrhoea	Cohort	×	✓	×	×	✓	×
Livingston et al. (2014) (30)	Coping strategies for carers of people with dementia	Randomized controlled trial (RCT)	✓	×	×	✓	×	✓
Bonell et al. (2015) (12)	Anti-bullying programme	RCT	×	✓	×	×	✓	✓
Freeman et al. (2015) (31)	Testing kits for Crohn’s disease	Review and model	×	✓	×	✓	✓	✓
Guthrie et al. (2015) (32)	Impact of NIHR HTA programme	Review	×	×	×	×	×	×
Michie et al. (2015) (33)	Taxonomy of behavioural change techniques	Methods paper	✓	✓	×	×	✓	✓
Richardson et al. (2015) (34)	Screening for psychological and mental health issues in young people	Review and model	×	✓	×	×	×	✓
Bailey et al. (2016) (35)	Web-based sexual health app	RCT	✓	✓	×	×	×	✓
Field et al. (2016) (36)	Lung cancer screening	RCT	×	✓	×	✓	✓	✓
Fortnum et al. (2016) (37)	School-entry hearing test screening	Model	×	✓	×	✓	✓	×
Freeman et al. (2016) (38)	My5-FU assay monitoring in chemotherapy patients	RCT	×	✓	×	✓	×	×
Jackson et al. (2016) (39)	Uptake of immunization in travelling and gypsy communities	Qualitative interview	×	✓	×	×	✓	✓
Parry et al. (2016) (40)	Cognitive behaviour therapy for fear of falling in older people	RCT	×	✓	×	×	✓	✓
Paton et al. (2016) (41)	Improving outcomes for people with mental health crises	Review	✓	✓	×	×	×	×
Tufail et al. (2016) (42)	Diabetic retinopathy image assessment software	Cohort study	×	×	×	✓	✓	×
Whitaker et al. (2016) (13)	Programmes to reduce unintended pregnancies	Review and model	×	✓	×	✓	✓	✓
Birrell et al. (2017) (43)	Real-time influenza modelling	Model	×	×	×	×	×	×
Flowers et al. (2017) (44)	Behavioural change programme	Review	×	✓	×	×	✓	×
Melendez-Torres et al. (2017) (45)	Beta-interferon and glatiramer acetate for treating multiple sclerosis	Review and model	×	×	×	×	×	×
Snooks et al. (2017) (46)	Assessment of protocols for older people following a fall	RCT	×	✓	×	✓	×	×
Soomro et al. (2017) (47)	Surveillance for small renal tumours	RCT	✓	✓	×	×	×	×
Thomas et al. (2017) (48)	Breathing retraining exercises in asthma patients	RCT	✓	✓	×	×	×	✓
Watson et al. (2017) (49)	Family and social network intervention for young people who misuse drugs and alcohol	RCT	×	✓	×	×	✓	✓
Waugh et al. (2017) (50)	Spot protein-creatinine ratio and spot albumin-creatinine ratio to assess preeclampsia	Model	×	×	×	✓	×	✓
Welton et al. (2017) (51)	Screening strategies for atrial fibrillation	Review and model	×	✓	×	✓	✓	✓
Williams et al. (2017) (52)	Timing of surgical intervention for developmental dysplasia of the hip	RCT	×	✓	×	×	×	×
Avenell et al. (2018) (53)	Bariatric surgery	Review and model	×	✓	×	✓	×	×
House et al. (2018) (54)	Self-management in adults with type 2 diabetes and learning difficulties	RCT	×	✓	×	✓	✓	×
McClurg et al. (2018) (55)	Abdominal massage for neurogenic bowel dysfunction in multiple sclerosis	RCT	✓	✓	×	×	✓	✓
Paleri et al. (2018) (56)	Gastronomy tube feeding in chemoradiation patients	RCT	✓	✓	×	×	✓	✓
Peron et al. (2018) (57)	Colposcopy techniques for assessing cervical abnormalities	Review and model	×	×	×	✓	×	×
Richards et al. (2018) (58)	Psychological care in cardiac rehabilitation	RCT	✓	✓	✓	✓	✓	✓
Saramago et al. (2018) (59)	Prenatal testing foetal rhesus D status	Review and model	✓	✓	×	✓	✓	×
Seguin et al. (2018) (15)	Self-sampling kits for HIV testing	Review, cohort study, and model	✓	✓	✓	✓	✓	×
Allan et al. (2019) (60)	An intervention to improve outcomes in falls in dementia	RCT	✓	✓	×	✓	×	✓
Griffin et al. (2019) (61)	Management of fracture of the distal femur	RCT	✓	✓	×	✓	×	×
James-Roberts et al. (2019) (62)	Support package for excessively crying infants	RCT	×	✓	×	✓	×	✓
Madan et al. (2019) (63)	Behaviour change package to prevent hand dermatitis	RCT	✓	✓	×	✓	×	×
Simmonds et al. (2019) (64)	Imaging or detection of osteomyelitis	Review	×	×	×	×	×	×
Francis et al. (2020) (65)	Management of acute exacerbations of chronic obstructive pulmonary disease	RCT	✓	✓	✓	✓	✓	✓
Surr et al. (2020) (66)	Dementia care mapping to reduce agitation in care home residents	RCT	✓	✓	×	✓	✓	✓

## Results

**Fig. 1:**
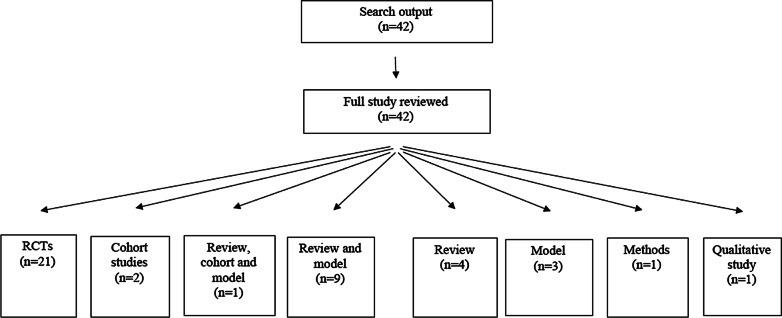
PRISMA [Preferred Reporting Items for Systematic Reviews and Meta-Analyses] 2009 flow diagram

Four hundred and forty-five studies were identified in the NIHR HTA programme between September 2014 and September 2020. Forty-two (9%) of these studies included the word “implementation” in the title or abstract (Fig. 1).

The extent to which implementation was formally incorporated in the analysis, and how implementation was defined, varied among studies. No studies were excluded from the review. Seven themes which are not included in the Proctor et al. (2012) checklist [study type, process evaluation (“the process of understanding the functioning of an intervention, by examining implementation, mechanisms of impact, and contextual factors” [20]), barriers and facilitators, quantitative evaluation of implementation, economic evaluation, recommendations, and future work] were of particular relevance to our research question (Table 3).

### Study type and setting

Twenty-one (50%) studies were either based on randomized controlled trials (RCTs) or pilot RCTs, while the remaining 50% of studies were a mix of cohort studies, modelling studies, or literature reviews (one monograph was a methods study) (Table 3). Twenty-eight studies assessed an intervention which applied to a clinical setting, while the remaining studies involved a population intervention (Table 3). Ten of the studies included discussed “setting” and the extent to which there was a readiness to adopt a new intervention or capacity to change (Table 2).

### Process evaluation, stakeholder engagement, and barriers and facilitators

A full process evaluation was included within 15 of the studies (Table 3). Two studies included a conceptual model of the decision problem [21, 22] (Table 3). In evaluating an intervention aimed at reducing bullying and aggression in schools, Bonnell et al. [23] used a conceptual model to map out and disaggregate the relationships between intervention inputs and how these were mediated via behavioural change and environmental change to produce health outcomes. A justification for the choice of interventions being considered was given in every monograph reviewed (Table 2).

Twenty-nine of the studies included reported engaging with stakeholders during their study (Table 2). Thirty-four studies included a discussion on barriers and facilitators to implementing an intervention (Table 3). This was the most common method by which implementation was considered within the studies included in this review.

### Quantitative evaluation of implementation and economic evaluation

Twenty-three of the studies included an economic evaluation (Table 3). Three studies included the use of quantitative data from a process evaluation to address implementation within their economic evaluation [24–26] (Table 3). For example, Richards [26] used semi-structured interviews to elicit data on nurse time required to undertake psychological care alongside cardiac rehabilitation in a pilot RCT. These data were then used to estimate the cost of nurse time.

Francis et al. [25] undertook a multicentre RCT in 86 general practitioner (GP) offices to evaluate the use of point-of-care testing to guide the management of antibiotic prescriptions in patients with chronic obstructive pulmonary disease. Embedded within the trial, a process evaluation found that staff time and initial training and equipment costs were a potential barrier to implementation of testing in routine practice. These findings were included within the economic evaluation. The results of the economic evaluation were then presented in terms of cost-effectiveness (cost required to reduce the number of people consuming at least one dose of antibiotics by 1%), cost-utility (cost per quality-adjusted life-year [QALY]), and cost consequence (where costs were presented alongside clinical outcomes in tabular form to allow decision-makers to determine for themselves the value they place on each clinical outcome in the trial).

Seguin et al. undertook a mixed-methods study to evaluate the use of self-sample kits for increasing HIV testing among black Africans in the United Kingdom [24]. The qualitative information collected in the process evaluation was used to guide the base case economic evaluation and to inform where sensitivity analyses were required around their assumptions. For example, the qualitative evaluation highlighted challenges in estimating the time required for a nurse to explain the intervention to the patient, since the majority of the appointment was spent explaining the study, obtaining consent, and recording baseline characteristics. As a result, reliable data on the time required to explain how to use the self-sample kit was not obtained. This informed the sensitivity analysis of the cost-effectiveness results, where nurse time and cost required to explain the intervention were varied and demonstrated that this was highly unlikely to impact on the overall cost-effectiveness results.

In all three of these studies, this involved the inclusion of additional implementation-related costs of delivering an intervention. No studies evaluated the relative cost-effectiveness of alternative implementation strategies.

Implementation was typically considered the remit of the qualitative researchers only, taking the form of a separate chapter which was then considered in the discussion section alongside the primary study results. Hence, there was little to no consideration of how implementation would impact on the economics of the intervention. For example, Little et al. undertook an RCT to investigate streptococcal management in primary care which included a nested qualitative study and economic evaluation [27]. The qualitative study gathered GP, nurse, and patient views regarding the challenges associated with the use of streptococcal tests in primary care. However, these data were not then used to consider the economics of alternative implementation strategies or to test the robustness of their results to alternative assumptions regarding implementation.

### Clear recommendations for implementation and future work

No studies included within the review specified implementation as a primary objective of their study. However, 23 of the studies referred to implementation within their specification of the study objectives as an issue for consideration. Thirty-three studies considered implementation in their discussion section only. Twenty-one of the studies included provided clear recommendations on implementation (Table 3). For example, Whitaker et al. (2016) suggested that future economic evaluations of interventions to reduce unwanted pregnancies in teenagers adopt a “multi-agency perspective”, due to the potential cost impact of interventions on not only health, but social care providers also [22]. Surr et al. [28] evaluated the use of dementia care mapping (DCM) to reduce agitation and improve outcomes in care home residents with dementia. This was a pragmatic RCT of a complex intervention which included a process evaluation. The intervention was not found to be clinically effective or cost-effective. However, the process evaluation identified a significant challenge in adherence to the intervention—10% of care homes failed to participate at all in the intervention, and only 13% adhered to the intervention protocol over the required period to an acceptable level. Two homes withdrew from the study—one citing a personal belief in the ineffectiveness of the intervention. Therefore, recommendations included considering alternative modes of implementation which were not reliant on care home staff for delivery [28]. The discussion of implementation as an issue for “further research” was reported in 22 of the studies included (Table 3).

## Discussion

The extent to which implementation was formally considered varied among studies. Methods for examining implementation ranged from single stakeholder engagement events to the more comprehensive process evaluation. There was no obvious pattern as to whether approaches to implementation had evolved over recent years. Approximately half of the studies included an economic evaluation. However, it was uncommon for the economic analyses to incorporate issues relating to implementation. Where issues relating to implementation *were* including in the economic evaluation, this was limited to additional costs only. Where implementation of an intervention was considered more generally, such as in the process evaluation utilized in the Surr et al. [28] study, they found that it was difficult to determine if the lack of effectiveness of the intervention was a result of an inherent lack of efficacy in the intervention itself or due to implementation challenges. This highlights the need to consider implementation alongside the evaluation of a health technology throughout the design and evaluation life cycle.

Current MRC guidance on developing and evaluating complex interventions stresses the importance of considering development, feasibility/piloting, evaluation, and implementation in a cyclical sequence. Specifically, they suggest involving stakeholders in the choice of question and design of the research to ensure relevance. They also suggest taking into account context, such that benefits and costs which are not captured in study can be incorporated into the analysis. Our review would suggest that this guidance is not consistently adhered to in HTA studies over the last 6 years (see *Stakeholders and Setting* within the Table 3). There is no obvious trend in terms of how studies have incorporated implementation issues over time. A potential reason for this lack of consistency is perhaps that, although guidance is provided by the MRC on *what* to include within an evaluation of a complex intervention, there is little guidance on *how* this should be included.

The Proctor et al. (2012) checklist provides a set of key issues which need to be considered when undertaking an implementation study. This review has assessed NIHR HTA studies over the last 6 years which have included implementation. Although the purpose of these studies was not explicitly to undertake an implementation study, it is worth considering to what extent they would be judged sufficient to undertake an implementation study based on the checklist suggested by Proctor et al. (2012). Our findings suggest that the studies identified in our review have not fully addressed implementation and that they need to go further. The necessary elements, such as team expertise, are often already available within the project team. What is required is guidance as to how quantitative and qualitative methods can be integrated, alongside early stakeholder engagement, so as to allow for implementation to be woven into every stage in the evaluation.

Methods for economic evaluation are well established for assessing the value for money of competing interventions, given a fixed budget constraint for the healthcare system. However, an intervention which appears highly cost-effective based on these cost-effectiveness methods as they are applied to simple interventions may no longer be cost-effective once the process of implementation is considered. This is partly due to the impact complex interventions can have on both other services within the same disease area (e.g. acute treatment versus rehabilitation, patient pathway, and organizational challenges, etc.) and also on non-health sectors (e.g. education, justice, defence, etc.). The need to consider how the costs and benefits of health technologies fall on difference sectors, and budgets, reinforces the need for economic evaluation which considers these trade-offs simultaneously. The question of whether costs “unrelated” to an intervention ought to be included within an economic evaluation remains a contentious issue [29]. For example, mechanical thrombectomy is a costly, but cost-effective, treatment available for patients with acute ischaemic stroke [30]. Should the initial fixed capital costs of the comprehensive stroke unit and staff training which is required to undertake this procedure be included within an economic evaluation or just the per-procedure variable costs? Drummond argues that if health benefits arising from an intervention are projected over an individual’s lifetime, then all healthcare costs should similarly be projected [31]. The recommended approach here would be to annuitize the initial capital cost over the useful life of the asset to produce an equivalent annual cost. However, we still have the challenge of how to capture healthcare costs attributable to different budget holders within a single economic evaluation. Indeed, Wildman et al. suggest that new funding models may be required to address the challenge of matching benefits and opportunity costs which fall on different sectors when implementing complex interventions [32].

The preferred measure for estimating clinical benefits from a health economic evaluation perspective is the QALY. However, the costs and benefits of competing healthcare interventions are not always sufficiently captured within a QALY outcome. This is particularly an issue when considering the implementation of a complex intervention, where multiple outcomes may be relevant to multiple stakeholders. Methods for economic evaluation which do not reply upon the QALY are available, including cost-effectiveness, cost consequence, multiple-criteria decision analysis (MCDA), and discrete choice experiments (DCEs). However, these methods are not without their limitations and have been discussed extensively elsewhere [33, 34].

In addition to the barriers imposed by implementation costs, and the problem of determining which outcomes ought to be considered, further barriers to implementation remain. These include issues relating to the design of the healthcare system and the political environment in which these decisions take place. Smith et al. suggest a range of solutions for addressing barriers to implementation which go beyond cost-effectiveness analysis [35]. These include the need to model and disaggregate a range of potential outcomes, depending on alternative implementation scenarios and system configurations; the use of qualitative and quantitative evaluation techniques; and the involvement of the public in the decision-making process.

While not utilized in any of the studies included in this review, existing methods are available for estimating the “value of implementation” within an economic evaluation [5, 6, 8]. These typically focus on either estimating the potential cost-effectiveness of alternative implementation strategies, the trade-off between directing resources towards further research or towards further implementation, or establishing a “break-even” level of implementation at which an intervention may be cost-effective. However, these methods do not consider the initial challenge of deciding what outcomes ought to be included when attempting to incorporate implementation issues into the economic evaluation of a complex intervention, nor how these outcomes should be evaluated. While useful, these methods tackle only a subset of the issues relating to implementation and are designed to be utilized *following* a cost-effectiveness analysis. We argue that we need to understand the potential challenges of implementation before we begin an economic evaluation so that these issues can be incorporated into the analysis.

More descriptive methods are also being developed to aid the economic evaluation of implementation. Anderson et al. (2016) advocate for a more “realist” approach to economic evaluation where, rather than a focus on “measurement”, the focus is on understanding what works, for whom, and in what circumstances [36, 37]. More recently, McMeekin et al. demonstrated the use of conceptual modelling alongside economic evaluation to explore the relationship between the disease, treatment, and other potential mediators which impact on the “success” of an intervention [38]. Both of these methodologies are contrasted with a more “black-box” approach to economic evaluation. Dopp et al. developed a framework for “mixed-method economic evaluation” in implementation science, highlighting the benefits to implementation science researchers from undertaking economic evaluations with context-specific information capable of informing the implementation process [7]. Each of these tools constitute another piece in the puzzle of integrating implementation within economic evaluation.

The implementation of health technologies is a complex problem. As such, it is unlikely that a single new methodology or perspective will address all the potential challenges associated with implementation. However, the incorporation of implementation issues into economic evaluation provides one route by which we can begin to address this problem and produce research which is more useful to decision-makers in a “real-world” setting.

Economic evaluations are increasingly incorporated within clinical trials with the aim of supporting the reimbursement decision-making process [39]. Analogous to the introduction of economic evaluation into clinical trials, we believe that economic evaluation should play a key role in guiding the process of implementing new interventions into routine practice.

THE NIHR HTA programme is only one funder of clinical research within the United Kingdom. An extensive search of other databases may have identified methods not included within our review. However, for pragmatic purposes, and due to the prominent role played by the NIHR HTA programme in setting the research agenda in the United Kingdom, we chose to limit our search to this database only. We limited our search to studies which included the word “implementation” within the title or abstract. There are a range of terms which may relate to implementation—e.g. capacity, acceptability, stakeholder, etc. However, as our aim was to capture how any of these issues relate specifically to the challenge of implementation, we think the choice to focus on this term is reasonable. It is a limitation of this study, but also a key point, that no guidance is available for evaluating how implementation has been incorporated within an HTA. To facilitate better implementation of research findings, further guidance will be required to help researchers decide how implementation ought to be considered within an economic evaluation from the outset and how these data should be analysed.

## Conclusion

There are currently a variety of approaches available to incorporate implementation within an HTA. While they all provide some insight into the issues surrounding implementation, they do not go far enough in terms of evaluation and giving recommendations on specific implementation strategies. Furthermore, the issues of economic evaluation and implementation are typically considered in isolation—with implementation factors only considered after the economic evaluation has taken place. Given the MRC’s warning that an evaluation which does not include a “proper consideration of the practical issues of implementation will result in weaker interventions”, this is a surprising finding [11].

Our review has demonstrated a lack of consistency in how implementation has been incorporated within NIHR HTA-funded research and, hence, a need for further guidance in this area. We argue that implementation ought to be considered early in the evaluation of a complex intervention. We further argue that implementation and economic evaluation ought to be integrated, such that an appreciation of the economic implications of implementation issues are considered iteratively throughout the evaluation process. We recommend a more strategic approach to considering implementation—plan ahead and collect data which will allow for a quantitative analysis, which can be supplemented by qualitative work to inform implementation. This can conveniently be done within the economic evaluation framework.

## Authors' information

The main author is a research associate in Health Economics and Health Technology Assessment within the Institute of Health and Wellbeing at the University of Glasgow. He is undertaking a PhD on methods for incorporating implementation within the economic evaluation of HTAs.

## Data Availability

All data generated or analysed during this study are included in this published article.
